# Supplementary material for: Placental malaria caused by Plasmodium vivax or P. falciparum in Colombia: Histopathology and mediators in placental processes
Source: PLoS One. 2022 Jan 25;17(1):e0263092. doi: 10.1371/journal.pone.0263092 (PMC8789140; doi:10.1371/journal.pone.0263092)
Supplement: S1 Text — (DOCX) [file pone.0263092.s001.docx]

**Supplementary material 1. Definition of histological events in the placenta**

| Event | Definition |
| --- | --- |
| Abruptio | Placental abruption hemorrhage; there is an accumulation of blood under the decidua and its dissection. Abruptio represents the rupture of incompletely remodeled spiral arteries due to ischemia-reperfusion or atherosis. It can present as placental abruption, marginal abruption, acute marginal abruption, or chronic (marginal) abruption (1). |
| Atherosis | Change in the spiral arteries of the decidua, specifically the thickening of the arterial endothelium. The arterioles of the placental bed show signs of fibrinoid necrosis and foam cells; these alterations are placental atherosis and are similar to the changes seen in the vessels of patients with atherosclerosis (2,3). |
| Decidual necrosis | Ischemic area with degenerative lesions in the decidua. In decidual necrosis due to infections (malaria, toxoplasmosis, listeriosis, mycoplasmosis, virosis, etc.), focal areas of necrosis with intense infiltration, neutrophils, thrombi inside decisive blood vessels, and intense bleeding are common. VCs can be very edematous and lacking blood vessels (4-6). |
| Villous edema | Abnormal accumulation of fluid in the CVs' stroma, characterized by the expansion or swelling of them and the presence of voids in the stroma. "The accumulation of fluid in the chorionic villus stroma is a poorly elucidated entity. During the second half of pregnancy, there are 13% placentas with hairy edema” (7,8). |
| Chorionic villus infarction | Ischemic necrosis of an organ (tissue death due to lack of blood and subsequently oxygen), usually due to obstruction of the arteries that supply it (9). Gestational arterial hypertension links the placenta with increased development of infarctions and lower organ weight (8-10). |
| Fibrinoid / ibrinous deposit or fibrin deposit | Accumulation of fibrin in the CVs' stroma or around the CVs (in the Intervillous space IVS) (9). The Intervillous space that separates the villi from each other is very variable and can be occupied by an eosinophilic material called fibrinoid (8). |
| Syncytial nodules or syncytial nodes or syncytial knots | From the second trimester of pregnancy, STB cells begin to cluster in compact nests on the VC's surface, leaving spaces devoid of syncytium; those cell clusters are syncytial nodes (11). The presence of syncytial nodes has a positive correlation with the time and severity of pregnancy hypertension and the presence of fibrin in the CVs (12). Exposure to hypoxia and hyperoxia or reactive oxygen species induces syncytial nodes' formation (4,13-15); these phenomena are common in malaria. In terms of placentas, the prevalence of syncytial nodes in CVs greater than 33% should be considered increased (8,16). |
| Chorionic villi | The chorion is the fetal membrane in direct contact with the uterus' endometrium and covers the chorionic sac. It is formed by the STB, the cytotrophoblast, and the extraembryonic mesoderm. VCs are cytotrophoblast cells that proliferate on the outer surface of the chorionic sac forming cellular clusters that project to the STB, all this at the end of the second week (4). The various kinds of CVs differ in caliber, stromal structure, morphology, and blood vessel number (8,17). The approximate constitution of the term placenta is 40-50% of terminal villi, 25% mature intermediate, 20-25% trunk villi, 5-10% immature intermediate, and less than 1% mesenchymal villi (15). The number of villi seen per high-powered field is significantly increased in both in active and treated malaria cases compared to non-malaria controls; there is a significant decrease in villous areas in active malaria-infected cases compared to both controls and treated malaria cases (18). |
| Capillaries | The mesoderm cells in the center of the tertiary CV begin to differentiate into small caliber capillaries that form arteriovenous capillary networks that constitute the tertiary CVs. At the end of the third week, blood begins to circulate through the CVs' capillaries (4). |
| Capillaries per chorionic villus (villous vascularity) | The distal VC's capillary network serves to supply fetal blood after several generations of branching of the vessels that extend from the umbilical cord. The lesions to the integrity of the placental capillary network, which occur in different situations, have consequences that present serious risks to the health of the fetus, the infant, and the adult (18-20). |
| Hemorrhage in IVS | Blood flow from the circulatory system, caused by the rupture of blood vessels. In this work, attention was focused on hemorrhages evaluated microscopically, which correspond to "small vessel rupture (fetomaternal hemorrhage)" (1). "When histology confirms that the bleeding is enclosed by an infarction, the term hematoma should be used" (9). |
| Thrombus in IVS | Blood clot as a result of bleeding (8). |
| Calcifications in IVS | Deposition of calcium salts in the tissue (8,21). |
| Villitis or intervillositis | We do not address the diagnosis of villitis or intervillositis, but we limit ourselves to quantify the amount of immune cells present and to compare the amounts found in the placentas without and with plasmodial infection. |
| Immune cells | Total cells in each zone (decidua, villus, and intercellular space). Immune cells have nuclei that are deeply or densely stained (chromatin is large and bulky) and almost fill the cells, with only a slight edge of cytoplasm around the nuclei. This cell can have a nucleus divided into two to five round or ovoid lobes connected with thin chains or small chromatin bands. Events associated with immune cells are those of villitis and intervillositis, whose definition or diagnostic criteria are currently highly problematic (22-24). |

Source: own elaboration with the cited references.

**References**

1. Redline RW. Classification of placental lesions. Am J Obstet Gynecol. 2015; 213(4 Suppl):S21-8.

2. Staff AC, Dechend R, Pijnenborg R: Learning from the placenta: acute atherosis and vascular remodeling in preeclampsia-novel aspects for atherosclerosis and future cardiovascular health. Hypertension*.* 2010, 56:1026-1034.

3. Reyna-Villasmil E, Santos-Bolívar J, Suárez-Torres I. Microparticles in pregnancy and preeclampsia. Rev Obstet Ginecol Venezuela*.* 2013; 73(4):268-276.

4. Botella-Llusiá J. La Placenta. Fisiología y Patología. Madrid-España: Díaz de Santos, 1992.

5. Fox H, Sebire NJ. Pathology of the Placenta-Major Problems in Pathology. 3rd edition. China: Saunders-Elsevier, 2007.

6. Baergen R: Manual of Pathology of the Human Placenta. 2nd ed. New York, 2011.

7. Castejón O, Ali S, Canache L. El edema de la vellosidad placentaria en los casos de muerte fetal. Gaceta Médica de Caracas*.* 2006; 114(4):291-299.

8. Castejón-S O, López-G A, Péres Ybarra L, Castejón-M O: Presencia de alteraciones histopatológicas en vellosidades placentarias normales en Maracay (Venezuela). Rev Colomb Obstet Ginecol. 2009; 60(3):237-246.

9. Khong TY, Mooney EE, Ariel I, Balmus NC, Boyd T, Brundler MA et al. Sampling and definitions of placental lesions. Amsterdam Placental Workshop Group Consensus Statement. Arch Pathol Lab Med. 2016; 140(7):698–713.

10. Prieto-Gómez R, Ottone N, Sandoval-Vásquez C, Saavedra A, Bianchi H: Morphoquantitative characteristics of free corial vellosities in normal births, with diabetes, hypertension and restriction of intrauterine growth. Int J Morphol*.* 2018; 36(2):551-556.

11. Coleman SJ, Gerza L, Jones CJ, Sibley CP, Aplin JD, Heazell AE: Syncytial nuclear aggregates in normal placenta show increased nuclear condensation, but apoptosis and cytoskeletal redistribution are uncommon. Placenta*.* 2013, 34:449-455.

12. Corrêa RR, Gilio DB, Cavellani CL, Paschoini MC, Oliveira FA, Peres LC, Reis MA, Teixeira VP, Castro EC: Placental morphometrical and histopathology changes in the different clinical presentations of hypertensive syndromes in pregnancy. Arch Gynecol Obstet*.* 2008, 277:201-206.

13. Heazell AE, Moll SJ, Jones CJ, Baker PN, Crocker IP: Formation of syncytial knots is increased by hyperoxia, hypoxia and reactive oxygen species. *Placenta.* 2007, 28 Suppl A:S33-40.

14. Scifres CM, Nelson DM: Intrauterine growth restriction, human placental development and trophoblast cell death. J Physiol*.* 2009, 587:3453-3458.

15. Apaza-Valencia J: Desarrollo placentario temprano: aspectos fisiopatológicos. Rev peru ginecol obstet. 2014; 60(2):131-140.

16. Loukeris K, Sela R, Baergen RN: Syncytial knots as a reflection of placental maturity: reference values for 20 to 40 weeks' gestational age. Pediatr Dev Pathol*.* 2010, 13:305-309.

17. Isaza-Mejía G, Yepes-Zapata G, Castaño M: Arquitectura histológica de la placenta humana al contraste de fases y aplicaciones clínicas. Rev Col Obst y Ginecol. 1971; 22(1).

18. Chaikitgosiyakul S, Rijken MJ, Muehlenbachs A, Lee SJ, Chaisri U, Viriyavejakul P et al. A morphometric and histological study of placental malaria shows significant changes to villous architecture in both *Plasmodium falciparum* and *Plasmodium vivax* infection. Malar J*.* 2014; 13:4.

19. Prieto-Gómez R, Matamala F, Rojas M: Características morfológicas y morfométricas de la placenta de término, en recién nacidos pequeños para la edad gestacional (PEG) en la ciudad de Temuco-Chile. Int J Morphol*.* 2008, 26:615-621.

20. Merz G, Schwenk V, Shah R, Salafia C, Necaise P, Joyce M, Villani T, Johnson M, Crider N: Three-dimensional Rendering and Analysis of Immunolabeled, Clarified Human Placental Villous Vascular Networks. J Vis Exp. 2018; (133):57099. doi: 10.3791/57099.

21. Castejón O, Belouche R, Morett V: El proceso de calcificación en la placenta humana. Gac méd Caracas. 1998, 106(4):496-501.

22. Sanguansermsri D, Pongcharoen S: Pregnancy immunology: decidual immune cells. Asian Pac J Allergy Immunol. 2008, 26:171-181.

23. Bulmer JN, Williams PJ, Lash GE: Immune cells in the placental bed. *Int J Dev Bio.l* 2010, 54:281-294.

24. Ortega-Pajares A, Rogerson SJ: The Rough Guide to Monocytes in Malaria Infection. Front Immunol. 2018, 9:2888.
